# The Reformed Public House

**Published:** 1924-01

**Authors:** D. C. Dering


### CORRESPONDENCE.

[Correspondence on all subjects is invited, but we cannot be responsible for the opinions expressed by our correspondents, who must give name and address as a guarantee of good faith, but not necessarily for publication. Correspondents are reminded that conciseness greatly facilitates early insertion.]

### The Reformed Public House.

*To the Editor of* The Hospital and Health Review.

Sir,—The evil of the present licensing system rests upon the fact that it has resulted in the creation of a huge drink trust which, through the tied house system, concentrates its energies mainly on the sale of beer.

I am anxious to see the public house conducted as a place for the supply of food and non-intoxicants as well as of alcoholic beverages on the lines of the People's Refreshment House Association. Frankly, I dislike the ordinary public house and consider its continued existence a disgrace to the nation. If the State owned the liquor trade, we could ensure the conduct of the public house on rational lines. At present it is only a money-making concern in the interests of brewery shareholders.

The question of drunkenness statistics does not enter into the matter so far as I am concerned.

D. C. Dering.

